# Supplementary figures and images for: Performance of a Finnish Diabetes Risk Score in detecting undiagnosed diabetes among Kenyans aged 18–69 years
Source: PLoS One. 2023 Apr 26;18(4):e0276858. doi: 10.1371/journal.pone.0276858 (PMC10132597; doi:10.1371/journal.pone.0276858)

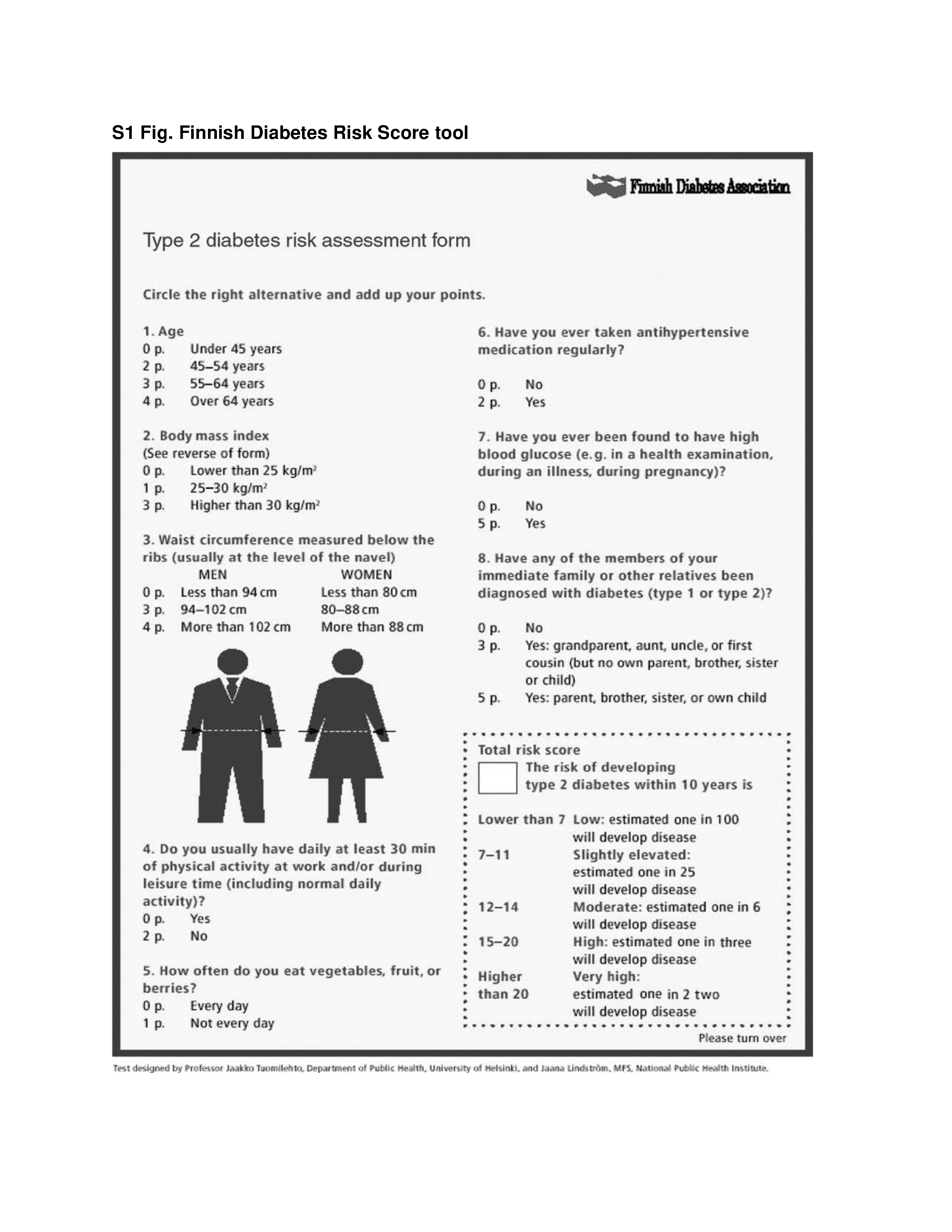

Supplement: S1 Fig — (TIFF) [file pone.0276858.s001.tiff]

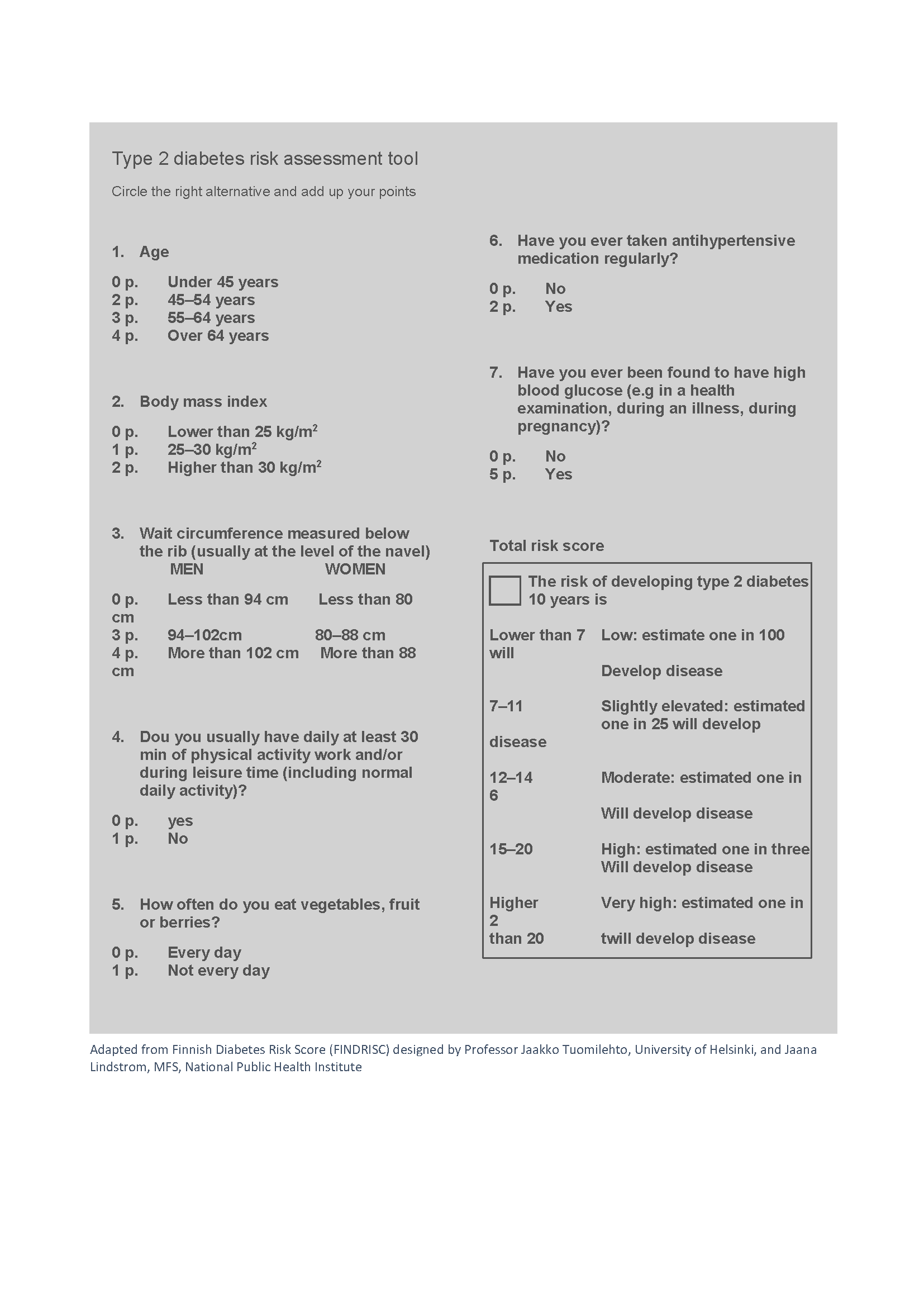

Supplement: S2 Fig — (TIFF) [file pone.0276858.s002.tiff]

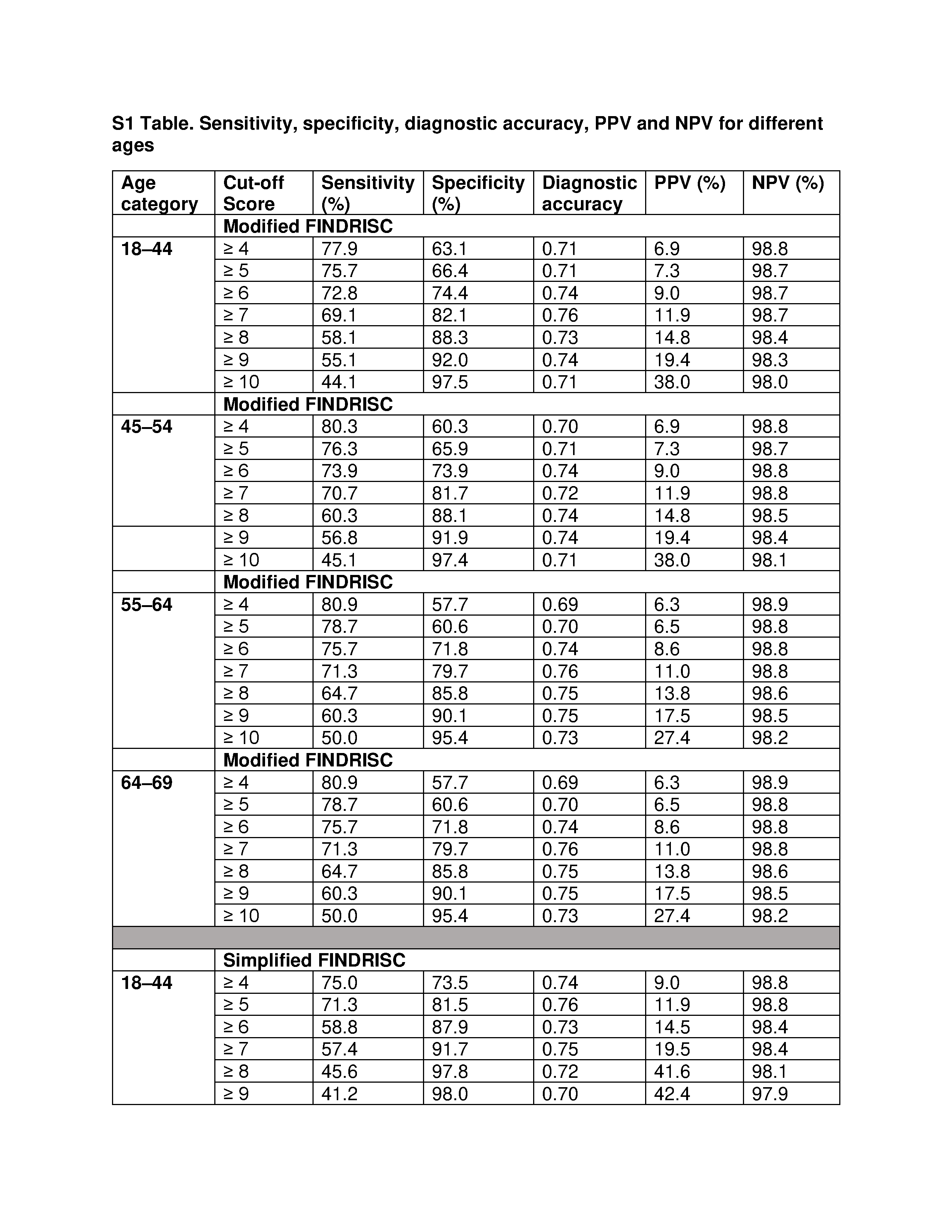

Supplement: S1 Table — (TIFF) [file pone.0276858.s003.tiff]
