# Supplementary material for: Performance of a Finnish Diabetes Risk Score in detecting undiagnosed diabetes among Kenyans aged 18–69 years
Source: PLoS One. 2023 Apr 26;18(4):e0276858. doi: 10.1371/journal.pone.0276858 (PMC10132597; doi:10.1371/journal.pone.0276858)
Supplement: Attachment — Submitted filename: Response to Reviewers Comments.docx [file pone.0276858.s004.docx]

*Dear Editor,*

*We thank you and the reviewers for their constructive and very relevant feedback. Thank you for the opportunity to revise this work. We have endeavored to address the concerns in the best way possible and we believe the revised version addresses all the concerns.*

*Below are the responses to the review questions.*

Reviewer #1: It is very interesting to use a risk score in pickup people at risk in low economic countries, the key is to evaluate a suitable existing engine tool and revise it to adopt in a specific population. You had done a valuable study, and found a simplifies risk score to cover most of the people at risk.

*Response:*

*We appreciate the comments and remarks from your review of our work, and we thank you for the recommendation given.*

Reviewer #2: The theme of this paper is important and have huge potential benefit to the developing nations. The major issue is that the conclusion of this paper lack significant statistical support. I suggest the authors to significantly increase the sample size for statistically significant conclusion

*Response:*

*We appreciate the remark, comments, and observations from your review of our work. Thank you for raising the issues of concern to us, and for the suggestion proposed. Our response to this is as follows.*

*Whereas our study sample size (n= 4,027) is slightly less than one used in the original FINDRISK studies conducted in Finland i.e., the model development survey (n=4,746) in 1987, and model validation survey (n=4,615) in 1992, our study was based on a national 2015 Kenya STEPs survey data (http//aphrc.org/wp-content/uploads/2016/04/steps-Report-NCD-2015.pdf) and we believe it had sufficient power to produce generalizable findings. Additionally, our study findings of the modified FINDRISC (Area under the curve (AUC = 0.7481) and simplified FINDRISC (AUC = 0.7486) when compared with the original FINDRISC (AUC = 0.87) findings, were slightly lower but demonstrated statistically significant findings in detecting undiagnosed diabetes in the low-income population setting such as Kenya (p <0.05). When the simplified FINDRISC score tool was compared with the modified FINDRISC score tool at a cut-off score value of ≥9, there was indeed no statistical difference (p = 0.9118) in detecting undiagnosed diabetes in the Kenyan population setting as demonstrated in both table 3 (page 15 of our manuscript) and figure 2 of our manuscript attached as supplementary file) hence the need to choose a simplified version since it needs fewer variables. We believe our study’s statistical findings demonstrate significant findings, and indeed support the conclusion of our manuscript paper.*

*Whereas we appreciate the suggestion of increasing the sample size, our study, sample size (n=4027), was a secondary data analysis of a national survey sample size (n=4,500). When compared with the original FINRISK studies conducted in Finland then, with sample sizes (n=4,746) and (n=4615) respectively, we believe our study sample size was comparable.*
