# Supplementary material for: Performance of a Finnish Diabetes Risk Score in detecting undiagnosed diabetes among Kenyans aged 18–69 years
Source: PLoS One. 2023 Apr 26;18(4):e0276858. doi: 10.1371/journal.pone.0276858 (PMC10132597; doi:10.1371/journal.pone.0276858)
Supplement: Attachment — Submitted filename: Response Rebuttal to Reviewer Comments_3_8_2022.docx [file pone.0276858.s005.docx]

*Dear Editor,*

*We thank you and the reviewers for their constructive and very relevant feedback. Thank you for the opportunity to revise this work. We have endeavored to address the concerns in the best way possible and we believe the revised version addresses all the concerns.*

*Below are the responses to the review questions.*

Reviewer #3: General Comments
In this study, using a modified and simplified FINRISC to detect undiagnosed diabetes in 4027 Kenyans aged 18-69 years, the authors examined how correct this inference of being diabetic. They found that this screening was acceptable to Kenyans, as already reported in some countries. They concluded that the simplified FINDRISC can distinguish individuals in a more cost-effective way, with or without diabetes. Statistical analysis seems sound. The manuscript is well written.
I have the following concerns:

Specific comments
1.　Sensitivity, specificity, and diagnostic accuracy depend on the prevalence of diabetes and are also influenced by race and lifestyle. As the author discussed in the text, there are quite a few reports on the accuracy of FINRISC for diabetes screening, including reports from Africa. The results shown here are unattractive.

*Response: First of all, we thank you for accepting to review our manuscript. We are also grateful for the comments raised, and the concerns highlighted which we have addressed as sufficiently as we can. As you rightly stated, it has been well documented that sensitivity, specificity, and diagnostic accuracy depend on the prevalence of the disease (in our case, diabetes) and could also be influenced by race, lifestyle, and genetic predisposition. Whereas a highly sensitive and specific test will have a high diagnostic accuracy in a population with a high disease prevalence, the same test will have a considerably low diagnostic accuracy in a population with low disease prevalence, as the case may be when the entire population is screened, which is true in the case of our study. Moreover, for our study, the population is fairly homogeneous by race and so, the race would not be expected to play a significant role. However, we acknowledge the influence of lifestyle attributes. Increasingly, the burden of diabetes and other non-communicable diseases in middle-and low-income country settings continues to rise and, therefore, the need to validate already existing screening tools in these population settings.*

*Whereas we concur with your observation that a few FINDRISC validation studies have been conducted in the African setting, the results of a few of those studies conducted (*Azzouz, M., et al., 2014, Masconi, K., et al., 2015*,* Omech, B., et al., 2016) *were fairly comparable to the validation studies conducted across many settings in Europe (Manuscript references: 22, 41-46). The results of our study, therefore, add to the growing body of existing knowledge on the validation of the FINDRISC tool, and its potential application as a primary care diabetes screening tool in different population settings. Although the results of our study may seem unattractive, they are comparable to some validation studies conducted across various settings, and we believe they are still of added value to the existing body of knowledge.*

2.　The original Finnish diabetes risk score was applied to subjects aged 35-64 years who were not treated with anti-diabetic drugs and was prospectively followed up for ∼10 years on the onset of diabetes, but the subjects recruited in this study were 27 to 60 years old (median 35 years, younger than the original study, where subjects were getting older during the follow-up of ∼10 years). The method and subject were different from this study. The original study predicted new cases of diabetes during the follow-up of ∼10 years. The meaning is different from this study. The results obtained here cannot be compared to the original results.

*Response: Thank you for your observation and comment. Whereas we acknowledge the difference in the design methods and age of study subjects, there is little we can do about the changing demographic shifts in the African setting. We acknowledge that our study findings may not be comparable with the original Finnish diabetes risk score study findings on the basis of the differences in study design since our study was of a cross-sectional design and aimed at predicting current diabetes. We, therefore, agree that the results of our study do not necessarily demonstrate the prediction of future diabetes(prognostication) but rather the identification of those at increased risk of current diabetes(diagnostic) that would benefit from the invasive diagnostic intervention. We, also acknowledge that some FINDRISC validation studies (e.g., Saaristo, T., et al.,2005,* Omech, B., et al., *et al., 2016,* Soriguer, F., et al., *2012, etc.) were also cross-sectional and therefore our study is no exception.*

3.　Even If this screening contributes to the detection of diabetes, is its use really useful? Determination of HbA1c and blood glucose levels is essential for the diagnosis of diabetes. If you want to diagnose current diabetes instead of predicting future diabetes, you should determine them. Those costs are not high.

*Response:* *Thank you for this comment. We really appreciate your knowledge and expertise about diabetes however, we believe validation of the FINDRISC as a primary care diabetes screening tool in our context is absolutely useful. Whereas WHO recommends HbA1c and point of care determination of capillary blood glucose as essential for the diagnosis of diabetes, in the African setting, they are not only costly but invasive too, not readily available in lower-level health facilities except at the hospital (tertiary) level. WHO also recommends* a *Package of Essential Noncommunicable (PEN) Disease Interventions for primary healthcare in low-income settings (WHO PENS, 2011).*

4.　Line 210: The number of subjects is wrong. 137, not 136.

*Response: Thank you for this observation. We have revised the manuscript text to reflect the correct number of subjects as 137 (see manuscript results section page12 line 211).*

Reviewer #4: The sample size question previously asked has been adequately answered.
You could say in the discussion that your sample size, while limited by the prior collection before this analysis was undertaken, is still comparable to those in other studies such as the original study in Finland and the one in Philippines.

*Response: We highly appreciate your suggestion here. We have revised the discussion section, and incorporated your suggestion accordingly (see discussion section page 16, lines 271 – 274).*

I am a new reviewer and have not seen the paper before. I have no major concerns.
I do have some suggestions on improving the paper for the reader.

Introduction page 4 line 57 suggest change to "inconvenient"

*Response: We appreciate your suggestion. We have revised the manuscript text accordingly as reflected in both, the revised version of our manuscript with track changes and the unmarked version of our manuscript without tracked changes (see Introduction page 4 line 57).*

In the first sentence of the last paragraph of the introduction add that FINDRISC was originally developed and used to predict risk of developing T2DM but has also been used to detect diabetes References 26,27,28.

*Response: Thank you so much for the suggestion. We have revised the manuscript and incorporated your suggestion as reflected in both, the revised version of our manuscript with track changes, and the unmarked version of the manuscript without tracked changes (see Last paragraph of the introduction section page5 lines 70,71 and 72).*

Study design Suggest move "conducted between April and June 2015" to household survey "conducted between April and June 2015" AND designed to...

*Response: We appreciate your suggestion. Thank you so much. We have incorporated your suggestion as reflected in both, the revised version of our manuscript with track changes, and the unmarked version of the manuscript without tracked changes (see Materials and Methods section page 5, lines 81, 83 and 84).*

Definitions
Omitting the 2 hour OGTT glucose will miss some cases. That's inevitable in this analysis but should be noted in the limitations. See Diabetic Medicine 2000 17(10): 741-745

*Response: Thank you for this observation. We, for sure, agree with you that using FPG alone may to some extend underestimate the prevalence of diabetes. However, as you rightly put it, it was inevitable in this study analysis. We have noted this in the limitations section of our manuscript (see Strength and limitations section page 19, lines 357, 358, 361, and 362).*

Criteria for BMI and waist circumference. Are these the ones appropriate for this ethnic group. Please specify.

*Response: We appreciate your observation and concern. For this study analysis, we used the WHO recommendations (see WHO reports 1999, 2000b, and 2008) that are recommended for universal consideration across ethnicities. Although the IDF has provided recommendations that are not only sex-specific but also population and geography-specific (see IDF, 2006; Zimmet & Alberti, 2006), it was not specific about the African population. While we appreciate that there could be differences in population characteristics, there is paucity of information in regard to the African context, and whereas the WHO guideline recommendations may not be the appropriate guidelines for this study population setting, we found them most suitable considering that they are evidence-based. This may be an area of interest for future research.*

Original Finnish (FINDRISC) diabetes risk score
A score of 21 or more predicts a 50% likelihood of developing T2DM in the next 10 years. What score do they use as a cut-off for detecting DM which is what you are doing. Please include both these points here.

*Response: Thank you for this observation. While the original Finnish diabetes risk score predicts a 50% likelihood of developing T2DM in 10 years’ time, the validated tool in our study identifies those at high risk of having diabetes (more of screening) that would benefit from the invasive diagnostic procedures. The decision for the cut-off score is largely a trade-off between sensitivity, specificity, diagnostic accuracy, and the predictive values (PPV and NPV) as discussed in our manuscript. For our study, we used, and recommend a cut-off score of ≥ 7, predicting a 7.9% (PPV) likelihood of having diabetes and 98.7% (NPV) likelihood of having no diabetes. We are however aware of the likelihood of falsely identifying those with diabetes as not having diabetes and those without as having, which is true of any screening tool.*

Modified FINDRISC It says personal history of diabetes was collected while in the original FINDRISK it was a history of a high glucose. You should note this here. The same applies for simplified FINDRISC. Also table 1 has high blood glucose history. It needs to be very clear and the text and table should be consistent.

*Response: Thank you for highlighting this observation. The primary study actually collected data on the history of high blood glucose and FPG test value, and history of hypertension for the study participants recruited. We have taken note of the discrepancy in the manuscript text, and have revised it to ensure consistency of the manuscript text (see Statistical analyses section page 10, lines 184, 185, 196, and table 1 page 12).*

Table 3 has LR and text has LHR.

*Response: Thank you for highlighting this observation. We have revised the text in the table to read likelihood ratio (LHR) to ensure clarity and consistency of the manuscript text.*

Discussion Last paragraph suggest contribute to the quality-of-care standard.

*Response: We appreciate the suggestion, and have revised the last paragraph of the discussion section of the manuscript accordingly (see page 19, lines 345 and 346).*

Omitting the 2 hour OGTT glucose will miss some cases. That's inevitable in this analysis but should be noted in the limitations. See Diabetic Medicine 2000 17(10): 741-745

*Response: Thank you for this comment. It was repeated here, and we already addressed it above.*
